# Prostaglandin E2 in the Regulation of Water Transport in Renal Collecting Ducts

**DOI:** 10.3390/ijms18122539

**Published:** 2017-11-27

**Authors:** Yuyuan Li, Yuanyi Wei, Feng Zheng, Youfei Guan, Xiaoyan Zhang

**Affiliations:** 1 Advanced Institute for Medical Sciences, Dalian Medical University, Dalian 116044, China; liyuyuan831221@163.com (Y.L.); yywei83@163.com (Y.W.); fzheng63@163.com (F.Z.); 2 Department of Physiology and Pathophysiology, School of Basic Medical Sciences, Dalian Medical University, Dalian 116044, China; 3 AstraZeneca–Shenzhen University Joint Institute of Nephrology, Shenzhen University Health Science Center, Shenzhen 518060, China

**Keywords:** collecting ducts, water transport, arginine-vasopressin, prostaglandin E2, aquaporin-2, nephrogenic diabetes insipidus

## Abstract

The kidney plays a central role in the regulation of the body water balance. The process of targeting the water channel aquaporin-2 (AQP2) on the apical plasma membrane of the collecting duct (CD) principal cells is mainly regulated by the antidiuretic peptide hormone arginine vasopressin (AVP), which is responsible for the maintenance of water homeostasis. Recently, much attention has been focused on the local factors modulating renal water reabsorption by AQP2 in the collecting ducts, especially prostaglandin E2 (PGE_2_). PGE_2_ is a lipid mediator involved in a variety of physiological and pathophysiological processes in the kidney. The biological function of PGE_2_ is mainly mediated by four G-protein-coupled receptors, namely EP1-4, which couple to drive separate intracellular signaling pathways. Increasing evidence demonstrates that PGE_2_ is essential for renal water transport regulation via multiple mechanisms. Each EP receptor plays a unique role in regulating water reabsorption in renal collecting ducts. This brief review highlights the role of PGE_2_ in the regulation of water reabsorption and discusses the involvement of each EP receptor subtype in renal collecting duct. A better understanding of the role of PGE_2_ in renal water transport process may improve disease management strategies for water balance disorders, including nephrogenic diabetes insipidus.

## 1. Introduction

The maintenance of whole body water homeostasis is dependent on water intake stimulated by thirst and water excretion regulated mainly by the kidneys. Approximately 180 L of glomerular filtrate is produced in an adult human each day, 90% of which is constitutively reabsorbed in the proximal tubules (PTs) and descending loop of Henle. The reabsorption of remaining filtered water occurs primarily in renal collecting ducts (CDs), and is tightly regulated to meet bodily needs. The collecting duct is a critical renal tubular segment for the maintenance of water homeostasis and in the determination of the volume and concentration of the final urine. It has been well accepted that the central regulator of water reabsorption in the CD system is arginine-vasopressin (AVP), a circulating hormone also known as antidiuretic hormone (ADH) [1,2].

AVP is produced by the supraoptic and paraventricular nuclei of the hypothalamus, and released from the neurohypophysis into the blood in response to small increases in plasma osmolality (1–2%) or greater depletions in circulating volume (≥10%). It binds to the vasopressin V2 receptor (V2R), a Gs-protein-coupled receptor, on the basolateral membrane of the principal cells of cortical and medullary CD cells [3]. The binding of AVP to V2R triggers an increase in the intracellular cyclic adenosine monophosphate (cAMP) levels and then the activation of protein kinase A (PKA), which phosphorylates aquaporin-2 (AQP2) protein leading to a rapid redistribution of AQP2 from intracellular vesicles into the apical plasma membrane. Increase in intracellular cAMP levels also result in PKA-mediated phosphorylation and activation of cAMP response element-binding protein (CREB), which increases AQP2 expression by enhancing its gene transcription. Moreover, increased cAMP levels also cause the inhibition of RhoA, possibly by PKA-mediated phosphorylation, leading to a partial depolymerization of the actin cytoskeleton, which is believed to facilitate the apical targeting of AQP2 protein [4]. Recently, evidence has been emerged supporting a concept that AVP can increase AQP2 membrane targeting via a cAMP-independent mechanism [5]. It has been previously reported that binding of AVP to V2R is capable of increasing intracellular calcium concentration in an Epac-dependent manner, which results in acute increase of osmotic water permeability [6]. AVP can also increase medullary osmotic pressure gradient by upregulating urea transporter UT-A3 expression to facilitate water reabsorption in renal collecting ducts [7].

Originally, it was believed that the regulation of renal water transport was solely dependent on AVP. However, during the past decade, a variety of autocrine and/or paracrine factors have emerged as important modulators of water transport process in the CD, including prostaglandin E2 (PGE_2_). PGE_2_ is the major cyclooxygenated metabolites of arachidonic acid (AA) produced in the kidney and is synthesized at high rates throughout the nephron and collecting duct system [8]. A large body of evidence demonstrates that PGE_2_ is of critical importance in regulating collecting duct water reabsorption. In this brief review, we will highlight the role of PGE_2_ in collecting duct water transport and discuss potential mechanisms involved in this process.

## 2. PGE_2_ Biosynthesis in the Kidney

**Figure 1:**
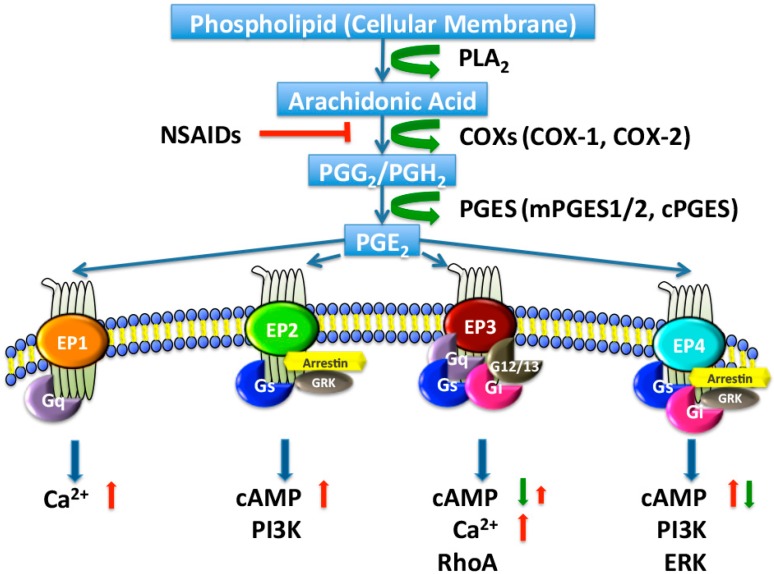
Biosynthesis and signaling pathways of prostaglandin E2 (PGE_2_). Arachidonic acid is released by phospholipase A2 (PLA_2_) from membrane phospholipids and metabolized by cyclooxygenase 1 (COX-1) or COX-2 to PGG_2_ and then PGH_2_ in a two-step reaction. COX activity is inhibited by nonsteroidal anti-inflammatory drugs (NSAIDs). PGH_2_ is relatively unstable and is enzymatically converted, by PGE synthase (PGES), to PGE_2_, which exerts its effects by binding to four specific G-protein-coupled receptors (GPCRs), designated EP1 to EP4. PGE_2_ interacts with one of four distinct EP receptors, each of which couples to distinct signaling pathways. In general, the activation of EP1 (coupled to Gq) increases intracellular Ca^2+^. The activation of EP3 (coupled to Gq) raises intracellular Ca^2+^ and/or (coupled to Gi) inhibits cyclic adenosine monophosphate (cAMP) production. The activation of EP2 or EP4 (both coupled to Gs) stimulates cAMP production. PI3K, PI3-kinase; ERK, extracellular regulated protein kinases; GRK, G-protein coupled receptor kinase; red arrows, increase; green arrows, decrease; red T bar, inhibition.

PGE_2_ is a major renal metabolite produced by a sequential process of three enzymatic reactions (Figure 1). The initial step of this metabolic pathway is the stimulus-induced release of AA from membrane phospholipids via a group of phospholipase A2 (PLA_2_) enzymes. The released AA is sequentially converted to unstable prostaglandin (PG) G_2_/PGH_2_ by cyclooxygenase 1 (COX-1) and COX-2, and then metabolized to PGE_2_ by a set of PGE synthases (PGESs).

### 2.1. Cyclooxygenases

Three isoforms of COX including COX-1, COX-2 and COX-3, have been identified to date. COX-1 is constitutively expressed, responsible for maintaining basic physiological functions, such as regulation of activation and aggregation of platelets [9] and cytoprotection of the gastric mucosa [10]. In the kidney, COX-1 is localized in the glomerulus, afferent arteriole (AA), PT, cortical and medullary CD, renal medullary interstitial cells (RMICs), as well as vasa recta (VR) [11,12]. In contrast, COX-2 is an inducible enzyme, which appears to play a key role in pathophysiological states, especially during inflammation and tumorigenesis [10,13]. Renal COX-2 expression is highly abundant in the macula densa (MD), cortical thick ascending limb (cTAL) and RMICs [14,15], with low expression found in the glomerulus (G), PT, and CD [16]. COX-3 is a splice variant of COX-1 and also observed in rodent kidney; however, very little is known about its renal functions [17].

### 2.2. PGE Synthases

Currently, three distinct enzymes have been identified with the capacity to convert PGH_2_ to PGE_2_, including microsomal PGE synthase-1 (mPGES-1), mPGES-2, and cytosolic PGES (cPGES) [18]. In general, the expression of mPGES-1 is highly inducible in response to physiological or pathological stimuli and acts in concert with COX-2 for PGE_2_ generation. In contrast, mPGES-2 and cPGES are ubiquitously and constitutively expressed and are thought to be responsible for the baseline PGE_2_ production coupled with COX-1 preferentially [19]. Among them, mPGES-1 is co-localized with COX-1 in the cortical and medullary CDs and is co-expressed with COX-2 in MD and RMICs [20]. Low but detectable levels of mPGES-2 and cPGES expression are also evident in the kidney. It has been reported that mice disrupted for mPGES-1 gene, but not for mPGES-2 or cPGES gene, exhibit suppressed PGE_2_ synthesis, indicating that mPGES-1 may play a more dominant role in the kidney [21,22,23].

## 3. PGE_2_ Receptors in the Kidney

**Figure 2:**
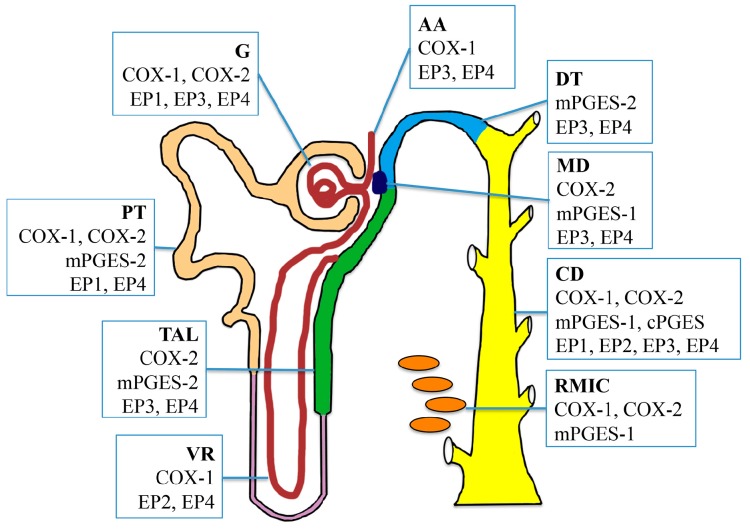
Intrarenal localization of cyclooxygenases (COXs), prostaglandin E (PGE) synthases, and PGE_2_ receptors EP1-4 throughout the nephron and collecting duct system. COX-1 is constitutively expressed in the glomerulus, afferent arteriole, proximal tubule, vasa recta, collecting duct, and medullary interstitial cells. COX-2 is expressed in the glomerulus, proximal tubule, thick ascending limb, macula densa, collecting duct, and medullary interstitial cells. Microsomal PGE synthase-1 (mPGES-1) is mainly localized in the macular densa, collecting duct and medullary interstitial cells. mPGES-2 is abundant in the proximal tubule, thick ascending limb, and distal tubule, while Microsomal PGE synthase-1 (mPGES-1) is expressed predominantly in the collecting ducts. EP1 is expressed in the collecting duct predominantly, and also in the glomerular podocytes and mesangial cells. EP2 has low levels of expression in the kidney, and it present only in the vasa recta and collecting duct. EP3 expression appears to be primarily localized in the thick ascending limb and collecting duct, with low expression in the glomerulus, afferent arteriole, distal tubule, and macula densa. EP4 is predominantly expressed in the glomerulus and collecting ducts, but also detected in other renal tubular segments. G, glomerulus; AA, afferent arteriole; PT, proximal tubule; TAL, thick ascending limb; VR, vasa recta; DT, distal tubule; MD, macula densa; CD, collecting duct; RMIC, renal medullary interstitial cell.

The diverse effects of PGE_2_ in the kidney are mediated through the activation of four G protein-coupled receptors (GPCRs), namely EP1-4. EP receptor subtypes also exhibit diverse renal localization and distinct intracellular signaling pathways, implying that each EP receptor may play a unique role in the kidney (Figure 2).

### 3.1. EP1 Receptor

The EP1 receptor acts predominantly via the Gq-protein. Activation of EP1 results in the stimulation of phospholipase C (PLC) and then protein kinase C (PKC), and ultimately an increase in intracellular calcium levels associated with modestly increased IP3 generation [24]. Among four EP receptors, EP1 has the least affinity for PGE_2_ [25]. Therefore, it is probably activated only when COX-2 expression is upregulated and PGE_2_ level is high. Within the kidney, EP1 is found to be expressed predominately in the CD [24]. Low levels of EP1 are also present in the glomerular podocytes, mesangial cells and PT cells [26,27,28].

### 3.2. EP2 Receptor

The EP2 receptor couples to G-stimulatory (Gαs) proteins, leading to increased cAMP levels in the cell, which results in the activation of PKA signaling. PKA directly phosphorylates and initiates the corresponding transactivation of transcription factors such as the CREB [29]. In addition, EP2-stimulated PKA activation can also activate the GSK3β/β-catenin pathway [30]. Unlike other EP receptors, EP2 expression in the kidney is low and its precise intrarenal localization remains incompletely characterized [31]. Some studies reported that EP2 was mainly found in vascular and interstitial compartments of the kidney. In addition, EP2 may be also constitutively present in the cortical collecting ducts [32,33]. However, we and others have previously reported that mice deficient for functional EP2 receptor develop salt-sensitive hypertension, supporting its important role in renal salt and water handling [34].

### 3.3. EP3 Receptor

To date, eight isoforms of EP3 have been reported in human, which result from alternative splicing of the EP3 gene and differ in the protein sequence of the intracellular C-terminal tail [35]. In mouse, alternative splicing only creates three EP3 splice isoforms, i.e., EP3α, β, and γ [36]. Almost all EP3 variants are primarily coupled to an inhibitory G-protein (Gi) and their activation results in the inhibition of adenylyl cyclase (AC) activity and a decrease in cAMP levels [37]. In addition, EP3γ is also found to be capable of coupling to Gs, leading to AC activation and increased cAMP production [36]. Although EP3β can also lead to AC superactivation, the underlying mechanism is different. EP3β increases cAMP levels mainly via the Gq/PLC/Ca^2+^ pathway in a lipid raft microdomain-dependent manner [38]. In addition to regulating AC, all three isoforms have the ability of intracellular Ca^2+^ mobilization via the Gi protein, resulting in the activation of the PLCβ isoform. Moreover, EP3 has been demonstrated to activate Rho kinase via G12/13 proteins [39]. In the kidney, EP3 receptor expression appears to be primarily localized in the TAL and CD, while precise intrarenal localization of its variants is largely unclear [40]. Low expression of EP3 is also present in the distal tubule (DT), glomerulus, AA, and MD [41].

### 3.4. EP4 Receptor

Similar to EP2, EP4 was initially characterized as a Gαs-coupled receptor, which also stimulates AC and cAMP production. However, the efficiency of the EP4 receptor coupling to the cAMP/PKA signaling pathway seems to be lower compared to EP2 [42]. EP4 has a second signaling pathway, in which it is coupled to a pertussis toxin-sensitive Gαi/0, leading to PI3-kinase (PI3K)-dependent activities [43]. Within the kidney, EP4 receptor mRNA is predominantly expressed in the afferent arterioles, glomerulus and collecting ducts [37], with low but detectable expression in almost all renal cell types, such as PT, TAL, as well as cortical CD [32,41].

## 4. Roles of Renal PGE_2_ in CD Water Transport

The effect of PGE_2_ on renal water absorption has been extensively investigated with conflicting conclusions. In some studies, PGE_2_ has been found to potently inhibit the effect of AVP on the osmotic water reabsorption in renal CD cells, consistent with the in vivo diuretic effects of PGE_2_ infusion. However, other reports demonstrate that PGE_2_ can also elicit a modest increase in water permeability in the absence of AVP. In the following section, we will discuss these findings with a focus on the role of each EP receptor in the regulation of collecting duct water reabsorption.

### 4.1. Role of the Key Enzymes for PGE_2_ Synthesis in CD Water Transport

Although COX-1 is constitutively expressed in the kidney, mice deficient for COX-1 appear to be healthy, with no obvious renal defects. In contrast, COX-2 seems to play an important role in regulating renal water transport. Mice with genetic disruption of COX-2 gene exhibit severe structural abnormalities and their kidneys appear pale and are smaller than those of the wild-type (WT) littermates. Anderson et al. [44] demonstrated that pretreatment of steroid-replaced hypophysectomized dogs with an unselective COX inhibitor, indomethacin, result in a significant increase in urinary osmolality in response to a fixed dose of AVP, indicating a direct effect of endogenous prostaglandins on the antidiuretic activity of vasopressin. This finding suggests that inhibition of endogenous prostaglandin biosynthesis appears to increase urine concentration. However, Baggaley et al. [45] reported an opposite effect, that nonsteroidal anti-inflammatory drugs (NSAIDs, including indomethacin) instead decrease AQP2 protein abundance, particularly during periods of dehydration. In addition, the homozygous deletion of COX-2 also results in the urine concentrating defect [46]. Although the reason for the discrepancy is currently uncertain, the use of different animal models with various hydration states may play a role. It appears clear that COX-2-derived prostaglandins downregulate transcellular water transporters including AQP2 and AQP3 in the collecting duct, since inhibition of COX-2 activity by targeted disruption or pharmacological blockade attenuates bilateral ureteral obstruction-induced AQP downregulation [47,48].

As discussed, expression of mPGES-1 is induced by proinflammatory stimuli, often in parallel with the induction of COX-2. Within the kidney, mPGES-1 predominates in the CD, a major renal tubular segment for the production and action of PGE_2_. In mPGES-1 knockout (KO) mice, AQP2 levels are increased at baseline and acute water loading-induced AQP2 reduction is blunted, suggesting an important role of mPGES-1 in provoking the diuretic response to acute overhydration [49]. In addition, following 24-h water deprivation, mPGES-1 KO mice promote urinary concentrating ability accompany with augmented increases in AQP2 expression [21]. Taken together, these results suggest that mPGES-1-deried PGE_2_ reduces urine concentrating ability through the suppression of renal medullary expression of AQP2. Although all three PGE synthases are capable of generating PGE_2_ in vitro, only deletion of mPGES-1 affects urine PGE_2_ levels in vivo. To date, the functional role of mPGES-2 and cPGES in renal physiology is still elusive.

### 4.2. Roles of EP1 and EP3 Receptors in CD Water Transport Regulation

The complicated effect of PGE_2_ on water permeability might be explained by which receptor is activated. The inhibitory effect of PGE_2_ on AVP-stimulated water reabsorption is possibly mediated by EP1 and/or EP3 receptors, both of which are expressed in the renal CD. EP1 couples to Gq protein and its activation by PGE_2_ increases intracellular calcium levels and inhibits water reabsorption in the microperfused CD [50], suggesting that renal EP1 receptor activation may contribute to the diuretic effects of PGE_2_. However, EP1 null (*EP1^−/−^*) mice seem healthy and fertile, without any impaired ability to excrete water [51]. In fact, EP1 receptor stimulation has never been shown to directly decrease AQP2 membrane targeting in the CD [33]. Interestingly, the urine osmolality of EP1-null (*EP1^−/−^*) mice cannot reach levels achieved by wild-type (WT) mice upon water derivation, but the expression and translocation of AQP2 in CD of *EP1^−/−^* mice appears equivalent to that of WT mice [52]. The urine concentrating defect observed in *EP1^−/−^* mice thus appears to be the result of blunted AVP production, since PGE_2_ can act on EP1 to promote AVP synthesis in response to acute water deprivation in the hypothalamus.

Renal EP3 is most recognized for its diuretic role in antagonizing AVP to inhibit AQP2 membrane targeting. This effect is commonly associated with its binding to a Gi protein, which attenuates cAMP production. Due to the existence of multiple EP3 gene splice variants in the CD, EP3 can also couple with G12/13 protein to activate the monomeric G protein Rho, which results in the inhibition of the depolymerization of the cytoskeleton and AQP2 translocation, thereby inhibiting water permeability [53]. Indomethacin, a non-selective inhibitor of endogenous PGE_2_ production, was demonstrated to increase urine osmolality in WT mice, but not in EP3 null (*EP3^−/−^*) mice [54]. Furthermore, activation by a selective EP3 agonist (sulprostone) significantly decreased, while inactivation by a specific EP3 antagonist (L-7981060) markedly increased, urine volume in rat [55]. This finding suggests that EP3 is involved in PGE_2_-modulated urinary concentrating ability. Surprisingly, *EP3^−/−^* mice exhibit similar urine-concentrating ability in response to AVP compared to wild-type mice [56]. Although the underlying mechanisms are unclear, it is speculated that the lack of EP3 may be compensated by other PGE_2_ receptors (such as the EP1 receptor) under basal conditions, with potential differences only emerging under pathological conditions.

### 4.3. Roles of EP2/EP4 Receptors in CD Water Transport Regulation

Similar to V2R, EP2 and EP4 are classified as Gs-coupled receptors as they are known to elevate levels of intracellular cAMP. In an inducible V2R gene knockout mouse model, EP4 selective agonist ONO-AE1-329 (ONO) can increase AQP2 levels and urine concentration [57]. Similarly, EP2 selective agonist butaprost alleviates the urinary concentrating defect caused by V2R antagonist in rats. Together, EP2 and EP4 both have the potential ability to increase urinary concentration in the absent of V2R. However, the underlying mechanism by which EP2 and EP4 promote urine concentration is different. For example, an EP2 receptor agonist (butaprost) increases cAMP levels and the phosphorylation of AQP2 at ser-269, whereas an EP4 agonist (CAY10580) has no effect on cAMP levels and ser-269 phosphorylation of AQP2 [33,57,58]. In addition, EP4 can couple to both Gs and Gi, whereas EP2 binds only to Gs. It is highly possible that EP4 may couple to both Gs and Gi to affect AQP2 gene transcription and protein phosphorylation. A recent study by Gao et al. [59] demonstrates that disruption of EP4 in the CD impaired urinary concentration via decreasing AQP2 abundance and apical membrane targeting. This study provides convincing evidence that EP4 can regulate the urine concentration independent of the AVP-V2R system. To date, whether EP2 may also promote urine concentration in the presence of AVP is unknown.

## 5. Interplay between the AVP and PGE_2_ Pathways in Optimizing CD Water Reabsorption

Increasing evidence suggests that interplay between the AVP and PGE_2_ pathways is critical for optimizing collecting duct water transport. It is well documented that AVP stimulates AC activity, increases cAMP production, and enhances the water permeability of the principal cell membrane. Additionally, it simultaneously stimulates phospholipase activity, which results in the release of AA from cell membrane and thus increases the rate of PGE_2_ biosynthesis. The stimulation of PGE_2_ synthesis by AVP can be inhibited by mepacrine that is an inhibitor of phospholipase activity, by the nonsteroidal anti-inflammatory agents that inhibit the COX, or by protein synthesis inhibitors that prevent hormone-stimulated activation of phospholipase. The stimulatory effect of AVP on PGE_2_ synthesis in the renal medulla is Ca^2+^-dependent and involves the activation of Ca^2+^-calmodilin-stimulated phospholipases. Interestingly, although AVP can increase both PGE_2_ and cAMP production in renal medulla, AVP-stimulated PGE_2_ production appears to be mediated by the V1 receptor (V1R), while AVP-induced cAMP production is the V2R-dependent [60]. In addition, a large body of evidence demonstrates that PGE_2_ can antagonize AVP action in renal collecting duct, possibly via multiple EP receptors and signaling pathways [61]. Cross talk occurring between the AVP and PGE_2_ pathways may fine-tune the expression and translocation of AQP2, therefore maintaining whole body water homeostasis.

## 6. Effects of Other Regulators on Collecting Duct PGE_2_ Biosynthesis

Besides PGE_2_, many other autocrine and paracrine agents, such as endothelin-1 (ET-1) and ATP/UTP, can decrease AVP-stimulated osmotic water permeability in the CD. Most of these agents can also regulate the production and release of PGE_2_. ET-1 has been reported to induce release of both PGE_2_ and stable metabolites of PGI_2_ or TXA_2_ in mesangial cells. Lee et al. reported that ET-1-induced PGE_2_ production can be attenuated by indomethacin in isolated diabetic rat glomeruli [62]. The mechanisms responsible for the induction of PGE_2_ synthesis may involve the activation of cytosolic PLA_2_ and the induction of COX-2 via the tyrosine phosphorylation signaling cascades. ATP can also stimulate a marked production and release of PGE_2_ through the activation of the P2Y2 receptor. This stimulation appears to be mediated by ATP-induced phospholipase activation, leading to increased levels of cAMP by the release of AA, and in turn, COX-derived PGE_2_. Sun et al. have demonstrated that P2Y2 receptor-mediated PGE_2_ release is dramatically increased in non-pathological polyuria induced by water loading [63]. This interaction is significantly enhanced in acquired nephrogenic diabetes insipidus (NDI), but blunted in dehydrated condition caused by water deprivation or chronic infusion of dDAVP (V2 receptor-specific analogue of AVP) in rats. Collectively, multiple local regulators may act in concert with PGE_2_ in controlling collecting duct water transport.

## 7. Complex Roles of PGE_2_ in Acquired Nephrogenic Diabetes Insipidus (NDI)

Nephrogenic diabetes insipidus (NDI) is an inability to concentrate urine due to impaired renal tubule response to AVP, leading to polyuria and polydipsia. It can be inherited or occur secondary to many clinical conditions that impair renal concentrating ability [64]. Congenital NDI is a rare disease in humans. In contrast to the rare inherited forms of NDI, acquired NDI is much more common. The most common cause of acquired NDI is chronic administration of lithium, the drug for the treatment of psychiatric diseases including bipolar disorders, schizoaffective disorders and depression. It has been reported that NDI patients have increased urinary excretion of PGE_2_. Consistently, in an animal study of lithium-induced NDI, the 24-h urinary excretion of PGE_2_ was also found to be significantly increased. Importantly, oral administration of indomethacin was found to be effective in reducing urine volume in a patient with lithium-induced NDI. Moreover, treatment of normal mice with a COX-2 inhibitor significantly attenuates lithium-induced NDI through the upregulation of AQP2 and Na-K-Cl cotransporter 2 (NKCC2), suggesting that NSAIDs or COX-2 inhibitors may represent potential therapeutic agents for the treatment of NDI [65].

Interestingly, lithium treatment does not result in polyuria and AQP2 downregulation in mice lacking mPGES-1, suggesting mPGES-1-derived PGE_2_ is a critical player in the pathogenesis of NDI. However, Kortenoeven et al. reported that indomethacin has no influence on the lithium-induced downregulation of AQP2, arguing against the role for PGE_2_ in lithium-induced polyuria [66]. This discrepancy may be explained by the fact that renal CD may alter its response to PGE_2_ in different physiological and pathophysiological settings, possibly through changing the abundance and profile of EP receptors.

## 8. Perspectives and Future Direction

**Figure 3:**
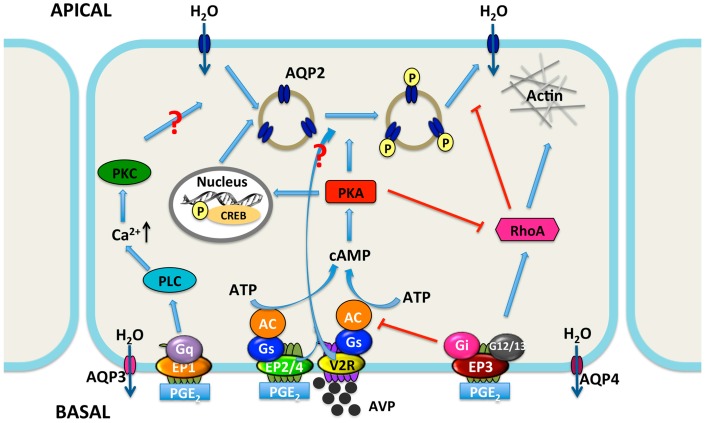
Proposed integrated model for water transport regulation by four PGE_2_ receptors in renal collecting duct cells. Arginine vasopressin (AVP) facilitates water reabsorption in renal collecting duct principal cells by binding to V2R in the basolateral membrane. This activates adenylyl cyclase (AC) via the G protein Gs, increases intracellular cAMP levels, and activates protein kinase A (PKA). Subsequently, AQP2 water channel is phosphorylated and translocated to the apical plasma membrane, rendering this membrane water permeability. In addition, PKA can decrease Rho activity by phosphorylating Rho, resulting in the depolymerization of F-actin and facilitating AQP2 translocation. PKA also increases AQP2 synthesis by phosphorylation of the cAMP-responsive element-binding (CREB) protein, which then binds to the AQP2 gene promoter. AVP-mediated AQP2 membrane targeting can be mediated via both cAMP-dependent and -independent pathways. PGE_2_ inhibits AVP-induced water reabsorption most likely mediated by EP1 and/or EP3 receptors. EP1 couples to Gq and induces the increase of intracellular Ca^2+^ levels followed by protein kinase C (PKC) activation, which counteracts AVP action by retrieving AQP2 from the plasma membrane. However, the role for collecting duct EP1 has not been fully established. The EP3 receptor couples to Gi, thereby inhibiting cAMP generation. Stimulation of the EP3 receptor by PGE_2_ also induces the activation of Rho, most probably via the G proteins G12/13. It promotes the formation F-actin, which hinders AQP2-bearing vesicles reaching the plasma membrane. However, activation of the EP2 and/or EP4 receptors may lead to elevate levels of intracellular cAMP via Gs. Water, entering the principal cell via AQP2, can exit from the cell via constitutively expressed AQP3 and AQP4. PLC, phospholipase C. APICAL, apical plasma membrane; BASAL, basolateral membrane; arrow, stimulation; red T bar, inhibition.

Antidiuretic hormone AVP is the key regulator of water reabsorption in the CD system and critically involved in the maintenance of water balance and stabilization of plasma osmolality. However, emerging evidence indicates that PGE_2_ can regulate water homeostasis either by interacting with AVP or by its receptors. The stimulatory effect of PGE_2_ on water permeability is most likely mediated by acting on EP4 and EP2 receptors, while the inhibitory effect of PGE_2_ on AVP-induced water reabsorption is possibly mediated by EP1 and/or EP3 receptors (Figure 3). Thus, the potential role of each EP receptor in collecting duct water transport regulation holds the promise of exploring the utility of EP receptor agonists and antagonists for the treatment of various water balance disorders, especially NDI. An attractive therapeutic approach for the treatment of NDI may involve the combination of the EP4 agonists and the EP1/3 antagonists. However, more studies are required to define the role of each EP receptor in water transport regulation and the molecular mechanisms involved in the action of PGE_2_ in renal collecting duct.

## Abbreviations

PT	proximal tubule
CD	collecting duct
AVP	arginine-vasopressin
ADH	antidiuretic hormone
V2R	vasopressin V2 receptor
cAMP	cyclic adenosine monophosphate
PKA	protein kinase A
AQP2	aquaporin-2
CREB	cAMP response element-binding protein
ET-1	endothelin-1
IMCD	inner medullary CD
PG	prostaglandin
AA	arachidonic acid
PLA_2_	phospholipase A2
COX	cyclooxygenase
PGES	PGE synthases
AA	afferent arteriole
RMIC	renal medullary interstitial cell
VR	vasa recta
MD	macula densa
TAL	thick ascending limb
G	glomerulus
DCT	distal convoluted tubule
GPCR	G protein-coupled receptor
PLC	phospholipase C
PKC	protein kinase C
AC	adenylyl cyclase
DT	distal tubule
PI3K	PI3-kinase
WT	Wide-type
NSAIDs	nonsteroidal anti-inflammatory drugs
NDI	nephrogenic diabetes insipidus
dDAVP	V2 receptor-specific analogue of AVP
NKCC2	Na-K-Cl cotransporter 2
